# Periappendiceal Abscess Masquerading as Pyosalpinx: A Case Report and Mini Literature Review of Acute Appendicitis Misdiagnosis

**DOI:** 10.7759/cureus.77364

**Published:** 2025-01-13

**Authors:** Anna Thanasa, Efthymia Thanasa, Gerasimos Kontogeorgis, Evangelos Kamaretsos, Ioannis Paraoulakis, Ioannis Thanasas

**Affiliations:** 1 Department of Health Sciences, Medical School, Aristotle University of Thessaloniki, Thessaloniki, GRC; 2 Department of Obstetrics and Gynecology, General Hospital of Trikala, Trikala, GRC; 3 Third Department of Obstetrics and Gynecology, University General Hospital "Attikon" Medical School, National and Kapodistrian University of Athens, Athens, GRC

**Keywords:** acute appendicitis, case report, computed tomography, misdiagnosis, pelvic inflammatory disease, surgical treatment, symptoms, ultrasound

## Abstract

A 17-year-old patient presented to the emergency department of the General Hospital of Trikala, Greece, reporting hypogastric pain accompanied by a fever of up to 38°C. The pain, progressively increasing in intensity, had been present for about a week, with the fever onset occurring 24 hours prior. Based on clinical examination, transvaginal ultrasound, and computed tomography findings, an incorrect diagnosis of pyosalpinx was made, and intravenous treatment with broad-spectrum antibiotics was initiated. However, the lack of improvement in the patient's clinical and laboratory findings after two days led to the decision to perform a laparotomy. Intraoperatively, a periappendiceal abscess was found, with a bilateral secondary extension of inflammation to the uterus, fallopian tubes, ovaries, and pelvic peritoneum. The inflamed appendix was resected from its retrocecal position, and the pelvic abscess was drained. The postoperative course was uneventful. This case report highlights an atypical presentation of acute appendicitis with abscess, which was preoperatively misdiagnosed as pyosalpinx. The main factors contributing to the misdiagnosis of acute appendicitis and the subsequent delay in medical care are discussed, emphasizing that early and accurate diagnosis is crucial in preventing adverse outcomes and ensuring effective treatment.

## Introduction

Acute appendicitis is a common surgical emergency in both children and adults and is one of the most frequent causes of acute abdominal pain, requiring early diagnosis and treatment. It typically occurs in the second or third decade of life and is estimated to affect approximately 1%-8% of children presenting to the emergency department with acute abdominal pain [1,2]. Acute appendicitis is more common in men (8.6%) compared to women, who have a lifetime risk of 6.7% [3]. Despite this, the percentage of appendectomy performed in females is higher (23.1% versus 12% in males) due to the misdiagnosis of pelvic inflammatory disease as acute appendicitis [4].

Pelvic inflammatory disease is usually an ascending polymicrobial infection of the lower genital tract, causing inflammation of the upper female reproductive organs, including the uterus, fallopian tubes, ovaries, and/or pelvic peritoneum [5]. The spread of inflammation and persistent infection within the fallopian tubes can lead to pus accumulation in the tubal lumen, resulting in the formation of a pyosalpinx [6]. Pelvic inflammatory disease is common in women of reproductive age, although its incidence cannot be accurately determined. The global prevalence is currently estimated to be between 4% and 12% [7,8]. Despite favorable clinical responses to contemporary antimicrobial treatments, pelvic inflammatory disease remains one of the leading causes of reproductive difficulties, significantly increasing the risk of long-term fertility-related complications [9].

This case report aims to highlight an atypical presentation of acute appendicitis with an abscess, which was preoperatively misdiagnosed as pyosalpinx. The diagnosis of acute appendicitis was made intraoperatively, 10 days after the onset of symptoms. The key factors contributing to the misdiagnosis of acute appendicitis and the subsequent delay in medical care are discussed, emphasizing that early and accurate diagnosis is crucial in preventing adverse outcomes and ensuring effective treatment.

## Case presentation

A 17-year-old patient presented (first visit) to the emergency department of the General Hospital of Trikala, Greece, reporting lower abdominal pain for approximately 12 hours. Associated symptoms, such as nausea, vomiting, diarrhea, constipation, and fever, were not reported. Her blood pressure was 110/70 mmHg, and her pulse rate was 75 beats per minute. No signs of peritoneal irritation were noted on abdominal examination. The pain was mild and localized in the hypogastric region, slightly above the symphysis pubis. McBurney's and Giordano's signs were negative. A transabdominal ultrasound revealed no abnormal findings in the intra-abdominal organs. Laboratory tests were within normal limits (Table 1).

**Table 1:** Blood test results of the patient from her first examination in the emergency department until her exit from the clinic ED: Emergency Department; Ht: Hematocrit; Hb: Hemoglobin; WBC: White Blood Cells; NEUT: Neutrophils; CRP: C-Reactive Protein; APTT: Activated Partial Thromboplastin Time; INR: International Normalized Ratio; Glu: Glucose; Cr: Creatinine; Na^+^: Sodium; K^+^: Potassium; TBIL: Total Bilirubin; SGOT: Serum Glutamic Oxaloacetic Transaminase; SGPT: Serum Glutamate Pyruvate Transaminase

Laboratory Tests	1st Examination in the ED	2nd Examination in the ED	Preoperative	2nd Postoperative Day	4th Postoperative Day	Normal Laboratory Values
Ht (%)	37.8	35.1	30.3	32.5	31.7	37.7–49.7
Hb (g/dL)	12.6	12.2	10.5	11.4	11.0	11.8–17.8
WBC (×10^3^/mL)	9.41	17.79	16.29	7.27	5.93	4–10.8
NEUT (%)	71.2	84.8	83.1	64.1	51.1	40–75
CRP (mg/dL)	0.3	15.65	15.01	3.89	0.96	0.5
APPT (seconds)	26.6	28.6	31.0	29.0	27.4	24.0–35.0
INR	0.98	1.22	1.34	1.27	1.01	0.8–1.2
Glu (mg/dL)	85	70	82	81	-	75–115
Cr (mg/dL)	0.55	0.80	0.77	0.65	-	0.40–1.10
Na^+ ^(mmol/L)	141.0	135.5	141.1	141.2	-	136–145
K^+ ^(mmol/L)	4.52	4.49	4.52	4.51	-	3.5–5.1
TBIL (mg/dL)	0.81	0.80	0.81	0.82	-	0–1.2
SGOT (IU/L)	18	19	14	15	-	5–33
SGPT (IU/L)	12	11	12	11	-	10–37

Abnormal findings were noted in the general urinalysis (80 leukocytes/high power field). A urine culture was recommended, yielding a negative result for pathogens. The result of the vaginal fluid culture was also negative. While awaiting the result of the urine culture, the patient was started on antibiotic treatment with oral cefuroxime (Zinadol 500 mg) at a dose of one tablet every 12 hours. Her medical and surgical history was unremarkable. Her body mass index was normal (BMI=23). The patient herself reported that the onset of sexual intercourse was four years ago. She did not respond clearly to the question regarding the total number of sexual partners. When asked if, in the past year, there had been more than one sexual partner, she responded affirmatively.

**Figure 1:**
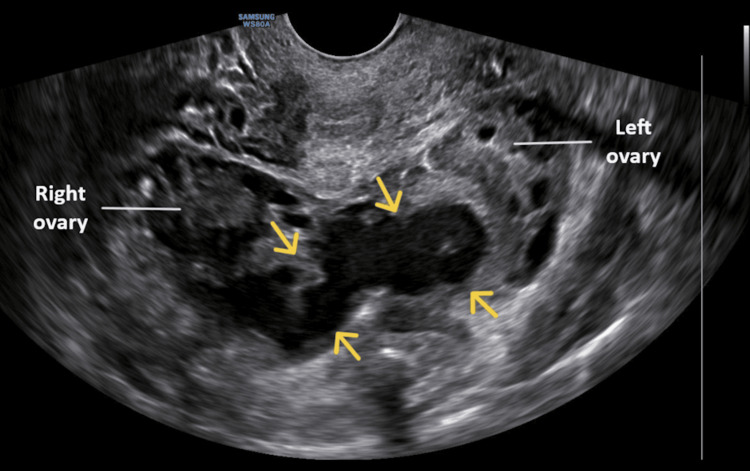
Transvaginal ultrasound imaging of the periappendiceal abscess misdiagnosed as pyosalpinx The presence of a tubular structure in the true pelvis (indicated by yellow arrows) led to the misdiagnosis of a pyosalpinx.

Without being asked for a follow-up, the patient reappeared (second visit) in the emergency department one week later. The pain in the lower abdomen had become more severe and was mainly located in the right iliac fossa. In addition, she reported a temperature of up to 38°C, which had started approximately 24 hours earlier. On gynecological examination, tenderness was noted upon applying pressure to the cervix. Transvaginal ultrasonography revealed the presence of a tubular formation in the true pelvis, indicating a possible pyosalpinx (Figure 1).

**Figure 2:**
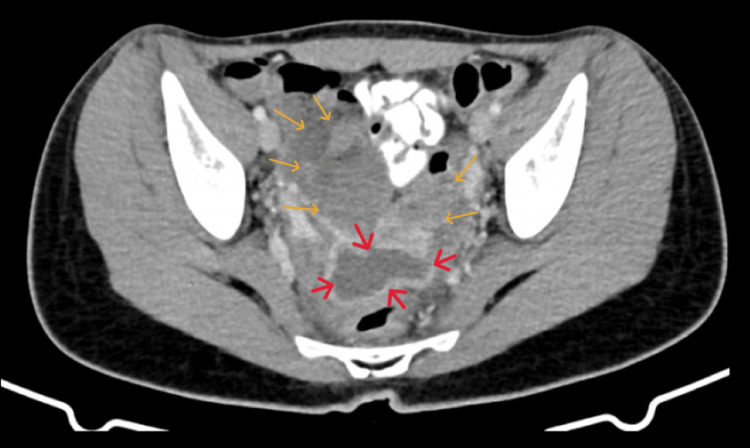
Computed tomography imaging of the periappendiceal abscess misdiagnosed as pyosalpinx An organized, thick-walled fluid collection with a spindle-shaped morphology (red arrows) is clearly visualized, along with increased opacification, diffuse fat exudation in the pelvis, and edematous imaging of the rectosigmoid (yellow arrows).

Computed tomography revealed an organized, thick-walled fluid collection with a spindle-like morphology, occupying the central part of the true pelvis between the ovaries. Additionally, increased opacification and fat exudation were noted diffusely in the lesser pelvis, along with edematous imaging of the rectosigmoid; these findings were considered more consistent with pelvic inflammatory disease (Figure 2).

The appendix was not clearly visualized, and inflammatory markers were elevated (Table 1). After admission to the clinic, intravenous antibiotic treatment with tazobactam (Tazocin 4.5 g) every six hours and tigecycline (Tygacil 50 mg) twice a day was started.

Two days later, the patient continued to have a temperature of 37.5°C. Inflammatory markers remained elevated (Table 1). The clinical symptoms and pelvic imaging findings were unchanged from those at admission. After extensive consultation with the patient and her family, surgical intervention with laparotomy was decided.

Intraoperatively, a periappendiceal abscess with a bilateral secondary extension of inflammation to the uterus, fallopian tubes, ovaries, and pelvic peritoneum was found. No hydrosalpinx, pyosalpinx, or tubo-ovarian abscess was identified. Pus culture was obtained from the periappendiceal abscess. The inflamed appendix was resected from its retrocecal position, and drainage of the pelvic abscess, along with thorough debridement of the pelvic region, was performed. An abdominal drain was placed. The postoperative course was uneventful, and the patient was discharged on the fourth postoperative day. One month after surgery, during clinical and transvaginal ultrasound examination, no signs of inflammation or other abnormal findings in the pelvic organs were observed.

## Discussion

Acute appendicitis, despite being a common surgical condition in adolescents and teenagers, can sometimes pose a serious diagnostic dilemma [10]. In a recent 2023 study, Weinberger et al. showed that 7.1% of patients with acute appendicitis are hospitalized in departments other than general surgery due to misdiagnosis. This results in a significant delay in surgical treatment and an increased risk of complicated appendicitis, leading to higher morbidity rates [11].

The clinical evaluation of acute appendicitis can be challenging, which may lead to diagnostic errors. Factors such as age, gender, race, and comorbidities significantly influence the likelihood of misdiagnosis. In patients younger than five years or older than 50 years, acute appendicitis often presents with atypical symptoms, increasing the risk of missed or delayed diagnosis [12]. Additionally, women are more likely to experience a misdiagnosis of acute appendicitis compared to men, largely due to pelvic inflammatory conditions, which can be mistaken for acute appendicitis in women of reproductive age [4]. These findings align with previous studies [4,12], and a more recent 2020 study by Mahajan et al. also reported that misdiagnosis of acute appendicitis is significantly associated with older age, female gender, White race, and a higher comorbidity index [13]. In our case, the patient was neither older than 50 nor younger than five years of age and had no coexisting conditions. However, the early onset of sexual activity and multiple sexual partners are among the risk factors that, along with clinical and imaging findings, led to the incorrect diagnosis of periappendiceal abscess as pelvic inflammatory disease with pyosalpinx formation. It is well known that young adulthood, having multiple sexual partners, and a history of previous pelvic inflammatory disease are major risk factors for the development of pelvic inflammatory disease [8].

Careful evaluation of symptoms and clinical signs is crucial to avoid the misdiagnosis of acute appendicitis. A recent study showed that patients with acute appendicitis who did not develop abdominal pain or developed abdominal pain alongside constipation were at high risk of misdiagnosis [13]. In general, the absence of symptoms such as abdominal pain, tenderness in the abdomen, nausea, vomiting, and leukocytosis can effectively exclude acute appendicitis with an accuracy of 98% [14]. In contrast, localized pain in the right iliac fossa, abdominal rigidity, and periumbilical pain radiating to the right lower quadrant are important clinical findings for diagnosing acute appendicitis in adults [15]. However, in sexually active women, pelvic inflammatory disease can present with similar symptoms to acute appendicitis, including lower abdominal pain, fever with or without chills, and rebound tenderness in the right iliac fossa. These symptoms are key features of the disease, and they can create a significant differential diagnostic challenge with appendicitis, leading to a delay in the correct diagnosis [16].

In our patient's case, both during her first visit to the emergency department and her second visit a week later (without a request for a follow-up), acute appendicitis was misdiagnosed. During the first visit, the lower abdominal pain was incorrectly attributed to dysuria and a possible urinary tract infection, a diagnosis based on the urinalysis findings and the absence of leukocytosis in the complete blood count. During the second visit, the periappendiceal abscess, visualized by transvaginal ultrasound and computed tomography, was mistakenly identified as pyosalpinx.

Another diagnostic pitfall that can lead to the misdiagnosis of acute appendicitis is the overreliance on laboratory tests and imaging results. Contemporary imaging modalities, although undoubtedly helpful in diagnosing acute appendicitis, should not replace a comprehensive clinical evaluation of the patient [17]. Compared to computed tomography and magnetic resonance imaging, ultrasonography is the preferred method for the diagnostic investigation of acute appendicitis, particularly in children [18]. In adults, computed tomography has demonstrated better sensitivity (76%-100%) and specificity (83%-100%) compared to ultrasound [19]. Cases of patients with pelvic inflammatory disease (tubo-ovarian abscess, pyosalpinx) misdiagnosed as acute appendicitis based on computed tomography findings have been described in the international literature [16,20]. Acute appendicitis in immunosuppressed patients is often difficult to diagnose and is associated with increased morbidity and mortality. It is very challenging for physicians to decide whether these patients should undergo appendectomy or expectant management [21].

Postoperative intra-abdominal abscess complicates 3%-25% of appendectomies, and the risk is highest following complicated appendicitis. A recent retrospective study shows that the technique of appendectomy does not appear to affect the incidence of intra-abdominal abscess, either in non-complicated or complicated appendicitis. However, laparoscopic appendectomy has the advantages of laparoscopic procedures, such as lower hospital stay and earlier return to activities, and should, therefore, be preferred for acute appendicitis [22]. In our patient, the presence of a pelvic abscess on imaging, combined with a history of multiple sexual partners, lower abdominal pain, and tenderness upon applying pressure to the cervix, led to the incorrect diagnosis of pyosalpinx rather than acute appendicitis. The cervical tenderness was due to a secondary extension of inflammation to the uterus, fallopian tubes, ovaries, and pelvic peritoneum.

## Conclusions

Acute appendicitis is a common surgical condition, and its diagnosis can often be challenging. Accurate and careful assessment of the overall clinical presentation, along with clear instructions for reassessing non-hospitalized patients, especially those with atypical symptoms, is crucial for making a correct diagnosis. Additionally, being aware of the risks of overreliance on laboratory and imaging test results is important in helping to avoid misdiagnosis. The goal is to prevent adverse outcomes and ensure timely and effective treatment.
